# Screening, characterization and optimization of potential dichlorodiphenyl trichloroethane (DDT) degrading fungi

**DOI:** 10.1016/j.heliyon.2024.e33289

**Published:** 2024-06-18

**Authors:** Girma Ebsa, Birhanu Gizaw, Mesele Admassie, Asnake Desalegn, Tesfaye Alemu

**Affiliations:** Department of Microbial, Cellular and Molecular Biology, Addis Ababa University, P. O. Box: 1176, Addis Ababa, Ethiopia

**Keywords:** 1,1-Dichloro-2,2-bis(p-chlorophenyl)ethane (DDD), 2,2-bis(p-chlorophenyl)-1,1-dichlorethylene (DDE), DDT, Degradation, GC-ECD, MALDI-TOF, Optimization

## Abstract

Dichlorodiphenyltrichloroethane is an organo-chlorine insecticide used for malaria and agricultural pest control, but it is the most persistent pollutant, endangering both human and environmental health. The primary aim of the research is to screen, characterize, and assess putative fungi that degrade DDT for mycoremediation. Samples of soil and wastewater were gathered from Addis Ababa, Koka, and Ziway. Fungi were isolated and purified using potato dextrose media. Matrix-Assisted Laser Desorption, Ionization, and Flight Duration The technique of mass spectrometry was employed to identify fungi. It was found that the finally selected isolate, AS1, was *Aspergillus niger*. Based on growth factor optimization at DDT concentrations (0, 3500, and 7000 ppm), temperatures (25, 30, and 35 °C), and pH levels (4, 7, and 10), the potential DDT-tolerant fungal isolates were investigated. A Box-Behnken experimental design was used to analyze and optimize fungal biomass and sporulation. The highest biomass (0.981 ± 0.22 g) and spore count (5.60 ± 0.32 log/mL) of *A. niger* were found through optimization assessment, and this fungus was chosen as a potential DDT-degrader. For DDT degradation investigations by *A. niger* in DDT-amended liquid media, gas chromatograph-electron capture detector technology was employed. DDT and its main metabolites, DDE and DDD, were eliminated from both media to the tune of 96–99 % at initial DDT concentrations of 1750, 3500, 5250, and 7000 ppm. In conclusion, it is a promising candidate for detoxifying and/or removing DDT and its breakdown products from contaminated environments.

## 1 Introduction

The development of agricultural and industrial activities has led to the production and release of numerous artificial and natural substances into the environment [[1], [2], [3]]. The chemical industry manufactures DDT as a synthetic chemical. Such synthetic chemicals are extremely persistent in the environment, as most microbes lack the necessary enzymes for their effective breakdown. However, certain microorganisms have evolved genetic mechanisms for degrading highly persistent DDT compounds [[4], [5], [6]]. DDT is still widely used as an organochlorine pesticide in Asia, Africa, and South America, although it has been banned in a many countries since the 1970s [[7], [8], [9]]. DDT is one of the dangerous persistent chemicals that have harmed biodiversity all over the world.

DDT is one of the dangerous persistent chemicals that have harmed biodiversity all over the world. Even though DDT was banned in many nations decades ago, research on it is still ongoing. The Stockholm Convention on POPs states that countries may manufacture and use DDT “when locally safe, effective, and affordable alternatives are not available” [10,11]. The Ethiopian government forbade the use of DDT in agriculture after that agreement, but not in the country's efforts to prevent malaria [[12], [13], [14]]. DDT and its residue found in the water, soil, and air endanger the ecosystem, especially the fauna and flora [[15], [16], [17]]. DDT residues persist in the ecosystem, mostly in the forms of DDT, DDE, and DDD. DDT and its metabolic products are lipophilic; they tend to accumulate in the fatty tissues of the species that eat them. DDT has been detected in high amounts in human tissues, including the brain, liver, placenta, blood plasma, and adipose tissue [[18], [19], [20]]. The majority of foods, especially processed foods, exhibit high amounts of DDT and its residue [[21], [22], [23], [24]]. From a global perspective, research conducted in Poland revealed that 53 agricultural soil samples have traces of DDT or its isomers and metabolites [25,26], whereas DDT was found in breast milk at a significant proportion (3.2 g/g fat) in India [19,24]. Ethiopia has been considered to have the largest accumulation of obsolete pesticides in Africa. It was estimated that there were 1500 tons of obsolete pesticides [27,28]. In the eastern part of Ethiopia, to control khat (Catha edulis) diseases, substantial quantities of DDT were applied. Because of this, consumers suffer serious health consequences as a result of DDT bioaccumulation [29]. According to reports by Ayele et al. [30], DDT pesticide residues are more common in Ethiopian food items. This suggests greater hazards for young children and babies, who are more vulnerable to pesticide toxicity because of their developmental stage and smaller body weight [29]. In Ethiopia, DDT and its metabolic products have been recognized in bird carcasses, plants, lakes, and human breast milk [30]. The level of DDT in Lake Ziway accounted for 92.3–98.6 % of all DDTs. More than 26 % of the birds exhibited p,p′-DDE, which has caused bird reproductive failure [[30], [31], [32]]. It exceeded the maximum residue limit (MRL = 0.02 g/g milk fat) set by FAO and WHO. One of the risk factors for breast cancer is the intake of DDT and its metabolites which may lead to the accumulation of primary products [[33], [34], [35], [36]].

The elimination of DDT and its metabolic products from ecosystem is given priority for environmental safety [6,37,38]. Microorganisms' bio-transformation of DDT is an economical and environmentally friendly method. This is opposed to physico-chemical approaches, which require large initial investments and generate difficult-to-manage waste throughout the process [[39], [40], [41]]. Several biological techniques, such as phytoremediation and bioremediation, were developed [[42], [43], [44]]. A wide range of environmental factors, including temperature, pH, water potential, and available nutrients, can also affect how quickly DDT degrade [19,45]. Nowadays, one of the most cutting-edge and economical methods for eliminating and detoxifying DDT from solid sludge, soil, sediments, groundwater contamination, and the environment as a whole is bioremediation [[46], [47], [48]]. Biorestoration of naturally existing microbes via bio-stimulation methods fosters the degradation of environmental contaminants [[49], [50], [51]]. Developing and changing metabolic pathways gives fungi a competitive advantage over other organisms by enabling them to adapt to changing environmental conditions and chemical stress [[52], [53], [54], [55]]. The metabolism of DDT may proceed via two pathways: dehydrochlorination, which happens in the presence of oxygen to form DDE. The second pathway is reductive dechlorination, which frequently happens in anaerobic conditions to form DDD. It is then changed to DDA, DDM, DBH, and DBP. The fact that certain fungi can convert DDT to DDE, DDD, DBH, and DBP when they are the only carbon source adds credibility to this. *Aspergillus niger* converted DBP marginally into 4-chloromethylbenzophenone and CBP. The production of CBP indicates a reductive dechlorination; this could be an intermediary impact of the DBP-to-BP conversion. After that, ring-cleavage processes totally degrade the BP [56,57,58,and6]]. *Pichia kluyveri FM012, Rhizopus arrhizus, Trichoderma hamatum, Pleurotus ostreatus, Fomitopsis pinicola, Pleurotus eryngii, Penicillium, Phlebia, Paecilomyces, Trichoderma, Allescheriella, Alternaria, Microsporum, Aspergillus,* etc. are a few of the fungi species that are known to degrade DDT. But Aspergillus species are the most widely used for degrading almost all types of pesticides [[59], [60], [61]]. These fungi play a vital role in the remediation of soil and water and are effective in converting dangerous organic compounds like DDT into harmless products [60]. Therefore, using indigenous fungi isolated from DDT-polluted environments as a DDT bioremediation tool offers a possible alternative to conventional methods [[62], [63], [64]].

Despite the fact that various researches have been carried out on the microbial degradation of DDT, the concentration has been conducted in small amounts, with the maximum quantities being 150 mL/L [65]. Ebsa et al. [60] conducted research on DDT degradation by using two fungi consortia which could transform DDT into its main metabolic products DDE and DDD sequentially. It reported capable of 7000 ppm initial amount of DDT. Nevertheless, there is a limited amount of research on DDT bioremediation using a single fungus in such a high concentration of DDT. As far as reviewing different works of literature, the majority of research done in Ethiopia is still centered on DDT residual analysis. Fungal-mediated bioremediation has been proposed as another solution to remove DDT from the ecosystems at a higher concentration of DDT. It has been suggested to use a statistically developed BBD-RSM to optimize the growth parameter for the efficient mycoremediation of this insecticides [66,67,and60]]. The capability of microbes to degrade DDT also depends on microbial sources for cleaning up DDT pollutants completely from the environment [68,69]. Therefore, the current study aims to screen, characterize, optimize growth parameters, and formulate bioinoculants of potential DDT-degrading fungal isolates for mycomediation purposes.

## 2 Materials and methods

### 2.1 Chemicals and standards

The purity standards for GC-ECD analysis grade DDT (98 %), DDD (99 %), and DDE (99 %) have been purchased from Taufkirchen Sigma-Aldrich Chemie Deutschland. Each of the DDT, DDD, and DDE standards was diluted to provide the GC-ECD (Gas Chromatography Electron Capture Detector) detection standards used for DDTr analysis. It was done with the DDT residues (DDTr = DDT, DDD, and DDE). DDT for analytical laboratory use was obtained from the pesticide manufacturer Adami Tulu in Ethiopia. The experiment employed only analytical-grade chemicals and solvents.

### 2.2 Description of the study area

**Fig. 1:**
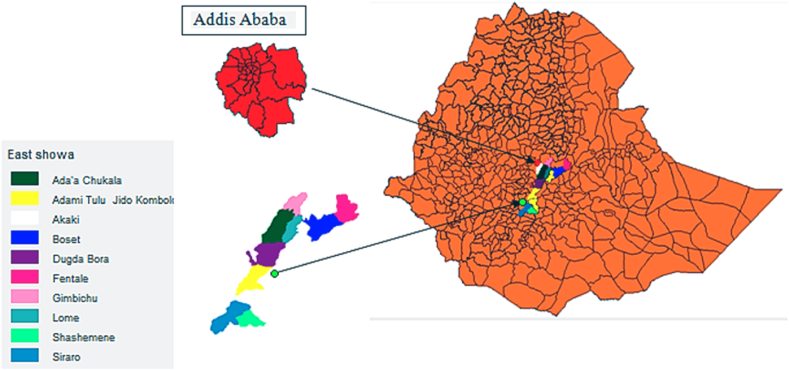
Map of study area.

Soil, wastewater, and effluent samples were collected from East Shewa, specifically Ziway, Koka, and Addis Ababa. The location under investigation was systematically chosen due to its agro-industrial waste disposal site, high levels of urban agricultural farming that used contaminated river water for irrigation, and a history of utilizing DDT for pest control. With geographic coordinates of 8° 9′ N, 38° 49′ E latitude, 38° 42′ 59.99″ E longitude, and an elevation of 1645.250 m above sea level, Ziway City is situated in Ethiopia. It lies 121 km south of Addis Ababa in the southern parts of the Oromia regional state, in the mid-rift valley. At a height of 1590 m above sea level, Lake Koka is situated in the Ethiopian Rift Valley (08°23′22″ N; 39°05′15″ E). From Addis Ababa, it is 90 km southeast. From a geographic perspective, Addis Ababa City is situated in Ethiopia at 9° 0′ 19.4436″ N latitude and 38° 45′ 48.9996″ E longitude. Its elevation is 2355 m above sea level (7726 feet), as illustrated in Fig. 1.

### 2.3 Sample Size, Type and Collection Method

With the help of a sterilized spatula, samples of agricultural farm soil, waste water, and effluent were collected from agro-industrial waste by plucking up to 5 cm of topsoil. The samples that were obtained were stored in a refrigerator in a sterile 45-mL Falcon tube. Using the stratified random sampling approach, a composite of 100 agricultural farm soil samples and 50 effluent wastewater samples was gathered. The Addis Ababa University Mycology Laboratory received all of the sample tubes, which each contained 25 mL of effluent wastewater and 25 g of soil. Then the samples' falcons’ tubes were refrigerated to +4 °C until processing.

### 2.4 Preparation of stock and working solution standards

DDT was dissolved in 0.1 mL of acetone to prepare the stock solution, which contained 20,000 parts per million. It was prepared in compliance with recommendations provided by the Organization for Economic Cooperation and Development [70]. Step-wise concentrations of 100 ppm, 500 ppm, 1000 ppm, 1500 ppm, 3000 ppm, 5000 ppm, 7000 ppm, and 10,000 ppm were achieved by dilution of the working solutions. All of them were refrigerated until more qualitative as well as quantitative studies were done. This was done in order to ascertain the maximum DDT concentration that could be applied to experiments that came after [71].

### 2.5 Experimental design

This study consists of three experiments: a resistance assay, an optimization assay, and a degradation assay. There were two factors involved in the DDT pesticide resistance assay. The amount of DDT concentration ranging from 100 ppm to 10,000 ppm and media type (DDT amended, non-amended, or control media) with 40 DDT-tolerant fungal species. The optimization tests were carried out using a four-factor design with three levels. This comprises two DDT concentrations (3500 ppm and 7000 ppm) and a control (no DDT), along with fungus species (AS1, AS2, and P1), temperature (25, 30, and 35), and pH (4, 7, and 10). In this study, response surface designs based on Box-Behnken produced 45 experimental runs, including one replication experiment. To assess the DDT degradation studies, a one-way ANOVA with a two-factor design was utilized. One replication experiment and a total of 27 experimental runs were generated by the ANOVA analysis. The two factors involved in this experiments are media type As1 + 1750ppmDDT, AS1 + 3500ppmDDT, AS1 + 5250ppmDDT, and AS1 + 7000ppmDDT agar media supplemented with minimal salt medium, (AS1 + 1750ppmDDT, AS1 + 3500ppmDDT, AS1 + 5250ppmDDT, and AS1 + 7000ppmDDT in PDB medium) and its metabolite analysis (DDE and DDD).

### 2.6 Culture medium enrichment and screening of DDT tolerant fungal isolates

In the DDT degradation experiment, minimal salt medium (MSM) composed the following gram per liter (g/L): 0.19 K2HPO4, 0.188 CaCl2, 0.198 KH2PO4, and 0.396 NH4 (SO4) 2, 0.4 MgSO4, and 1.65 NaCl were added to five 500-mL Erlenmeyer flasks modified from Nadeau et al. [72]. Every Erlenmeyer flasks contained 225 mL of pH 7.0 distilled water, 25 g of combined soil and wastewater sample, and 100 parts per million DDT. The mixture was then incubated for 15 days at 25 ± 2 °C in a New Brunswick Innova® 42 incubator shaker spinning at 120 rpm. In order to expose fungus to high DDT concentrations, the concentration of DDT was gradually increased, ranging from 500 ppm to 10,000 ppm. The isolation of fungi was performed using the dilution plate method (soil or effluent water ratio of 1:1000) [73]. Zero-point, 1-mL portions were transferred from each suspension into PDA growth media (PDA; Hi-Media, Mumbai, India) [73]. After a 5-day incubation period at 25 ± 2 °C on PDA, the isolates underwent purification and were identified using traditional taxonomic keys, taking into account both macro- and microscopic features [74,75]. In the Addis Ababa University mycology laboratory the pure isolates were stored at + 4 °C for further examination using a 50 % glycerol and 50 % PDB medium.

### 2.7 Identiﬁcation of fungal isolates

Following the optimization of the growth parameter, the fungal isolate AS1 was selected for the DDT degradation experiment. It is dependent on the ability to produce biomass and sporulate. Based on morphologic characteristics (macro and microscopic features) and molecular characterization using MALDI-TOF mass spectrometry analysis, this fungal species was identified [76].

#### 2.7.1 Morphological identification

The previously selected fungal isolate AS1 was subcultured onto potato-dextrose agar (PDA; Hi-Media, Mumbai, India). Using a cork borer with a 5 mm diameter, the culture was introduced in the center of each plate. After being inoculated, it was kept at 25 ± 2 °C as long as the 9-cm petri plate was completely covered. A ruler was used to measure the radial extension of the fungal mycelia every 24 h until the plates were covered. Using a Nikon D 5200 camera, macroscopic photos were taken and the following parameters were noted: mycelium colour, reveres side, growth state, margins, and elevation. Using the morphologic key to a visual atlas of soil and seed fungi, macroscopic identification was carried out [77]. On plate and slide cultures that were cultured on PDA for five days, microscopic observations were performed. The spore shape, hyphal nature, conidia, and conidiophores were noteworthy characteristics. Lactophenol Cotton Blue (LPCB) was used to mount the slides, and an Olympus CETI light microscope (Olympus Keyence International, Bedrijvenlaan, Belgium) was used for examination [77]. Microscopic photos were captured with a full-frame sensor camera (Canon 5Ds Mark III Thailand).

#### 2.7.2 Fungal identification by MALDI-TOF MS

A MALDITOF Biotyper Microflex LT mass spectrometer with a N2 (Bruker Daltonics, Bremen, Germany) laser tuned at 337 nm was used to analyze proteins [78]. Within a 2000–20,000 m/z range, the spectra were captured in positive ion mode using a 60 Hz laser. A total of 240 images taken from various locations on the metal plate were gathered for each spectrum. In addition, a steady flow rate of 70 L/min was maintained. The following values were set: 20 kV, 18.00 kV, 6 kV, and 150 ns for the acceleration voltage, extraction voltage, lens voltage, and delayed extraction time, respectively. The fungal isolates were cultured and ready for MALDI-TOF MS analysis according to the manufacturer's instructions (Bruker Corporation). The fungal isolates were grown on potato dextrose agar (Becton Dickinson GmbH, Heidelberg, Germany) and incubated at 25 ± 2 °C for three days, or until sufficient growth was observed. The mycelia were transferred into a microcentrifuge tube and centrifuged for 2 min at room temperature in order to identify the fungal isolates. With a pipette, the supernatant was extracted without coming into contact with the mycelial suspension. The pellet was allowed to air-dry at room temperature for 5 min. Subsequently, the pellet was vortexed on a benchtop at 13,000–15,000 rpm using 300 μl of water and 900 μl of 100 % ethanol (Sigma-Aldrich; Merck KGaA). The suspension was then centrifuged for 2 min at 13,000–15,000 rpm at room temperature to extract the supernatant. After letting the pellet air dry for 5 min, 25 μl of 70 % aqueous formic acid (Sigma-Aldrich; Merck KGaA) was vortexed with it. The pipette solution was then pushed up and down until the pellet was suspended in the same volume of acetonitrile. After that, the suspension was centrifuged for 2 min at 13,000–15,000 rpm. A polished steel target plate with 96 spots was filled with 1.0 μl of the supernatant and left to dry. For every sample, 1.0 μl of a saturated solution of MALDI-TOF MS matrix (a saturated solution of α-cyano-4-hydroxy-cinnamic acid in 50 % acetonitrile and 2.5 % trifluoroacetic acid) was applied. The mixture was then left to dry at room temperature (25–28 °C). FlexControl 3.1 and the Bruker filamentous fungus library 1.0 (Bruker Daltonics) were utilized to evaluate the mass spectra obtained using MALDI-TOF MS. The bacterial test standard (BTS; Bruker Daltonics) was utilized for the calibration of the spectrometer. Every sample was examined three times. *Aspergillus fumigatus* (ATCC® 3626TM; American Type Culture Collection) and *Aspergillus niger* (CBCB D2; National Center for Medical Culture Collections) reference strains were used for quality assurance prior to each assay. A score of 2.000 or higher indicated a species-level identification, 1.700–1.999 a genus-level identification, and <1.700 no identification [79,80].

### 2.8 Screening of DDT tolerant and significant variables using one way ANOVA statistical analysis

Fungal DDT tolerance screenings were conducted on petri plates containing solid culture medium, sterilized potato dextrose agar media, 50 % PDA +50 % DDT, and 100 % pure DDT + minimal salt (MS) supplemented with various DDT concentrations (100 ppm, 500 ppm, 1000 ppm, 1500 ppm, 3000 ppm, 5000 ppm, 7000, and 10,000 ppm). According to recently published studies by Al-Rashed et al. [49], the initial minimum DDT concentration was set at 100 ppm. The fungal maximum tolerable limit capacity was ascertained by testing the maximum DDT concentration of 10,000 ppm. The concentration of acetone in the last experiment medium was below the limit of 0.1 mL, as recommended by the Organization for Economic Cooperation and Development [65]. The final DDT concentrations (0, 3500, and 7000 ppm) were set for optimization studies. DDT-free media served as a control in the experiment. Fungal tolerance to DDTs was assessed by employing two indexes: one called the tolerance index (TI), which measures how much a fungus grows when DDTs are present or not based on the dry weights (DW) of fungal biomass as shown below in equation (1), and another called the sporulation index, which measures how much a fungus sporulates when DDTs are present or not based on sporulation capability [81,82].

(1) $$\%\;\text{Tolerance}\;\text{index}\;\left(\text{TI}\right)=\frac{\text{DW}\;\text{of}\;\text{treated}\;\text{mycelium}}{\text{DW}\;\text{of}\;\text{control}\;\text{mycelium}}\times 100$$

### 2.9 Fungal biomass production and sporulation determination

Regarding the assessment of fungal DDT tolerance, biomass production and sporulation were measured. The three-day-old mycelia fungal isolates of AS1, AS2, and P1 were transferred into a 500-mL Erlenmeyer flask containing 100 mL of a 1:1 ratio w/w PDB and DDT by employing a 5-mm-diameter cork borer (2X) or 10^10^ spores/mL. Potato dextrose agar was used as a control medium and incubated for 10 days. The mycelia growth of fungal isolates AS1, AS2, and P1 was filtrated from liquid cultures using a sterile membrane with a 0.22 μm pore size. The mycelia were placed in an oven to dry at 105 °C for 1 h, then at 80 °C for 24 h in order to determine the biomass [83]. The dried mycelia were weighed using an electronic balance (BA160 P). Dry biomass was expressed in grams, and a spore count was measured using a hemocytometer (Neubauer chamber, Taufkirchen, Germany). All of the experiments were carried out in triplicate.

### 2.10 Response surface methodology (RSM)-Based statistical optimization of biomass production and sporulation for the identification of significant factors

The Box-Behnken Design (BBD) is a statistical experimental design tool used to investigate the effect of each growth factor on fungal biomass production and sporulation. It is used to generate the experimental matrix [84]. Fungal isolates (AS1, AS2, and P1), DDT concentrations (0, 3500, and 7000 ppm), temperature (25, 30, and 35), and pH (4, 7, and 10) were the four independent variables used for optimization at three levels. In this study, the Box-Behnken family of response surface designs yields 45 experimental runs, including one replication experiment. Growing on DDT-amended (1:1 PDB + DDT at 3500 and 7000 ppm concentrations) and PDB as a control medium, the isolates AS1, AS2, and P1 were cultured. BBD was used for multiple regression analysis of quantitative data gathered from 45 experimental runs. The optimization study was carried out to evaluate and select significant variables for their optimal fungal biomass production and sporulation conditions. The optimization experiments were generated by Minitab 2019 software (Minitab Inc., State College, PA, USA).

### 2.11 Fungal DDT degradation assay by *A. niger*

After five days, *A. niger* mycelia were removed from PDA cultures by washing them with distilled water. Using a hemocytometer (Neubauer chamber, Taufkirchen, Germany) and an Olympus CETI light microscope (Olympus Keyence International, Bedrijvenlaan, Belgium), the amount of spores was adjusted. After adding DDT to the MSM, each liquid substrate was again suspended in a spore solution with an inoculum level of 10^8^ spores/mL [85]. The *A. niger* was pre-cultured in a 500-mL flask of DDT-amended MSM in a 100-mL volume at 27 ± 2 °C at 120 rpm for 15 days. To examine the effectiveness of *A. niger's* DDT degradation capacity. Four different DDT concentrations were investigated in two different media types: 1:1 ratio PDB + DDT and 100 % pure DDT supplemented with MSM. DDT-amended MSM with initial DDT levels of 1750, 3500, 5250, and 7000 ppm was used to inoculate *A. niger* in a 100-mL volume. Following the incubation period, sterile filter paper with a 0.22 μm pore size was used to separate the mycelia and their liquid medium. The filtrates were then centrifuged at 10,000 rpm. Fifty milliliters of the supernatant were taken for GC-ECD analysis after it had been diluted to 25, 50, 75, 100, and 125 ppb using sterile distilled water. There was a stepwise QueChERS (AOAC2007.01 or EN15663) extraction and clean-up method used for direct injection of the sample into the GC-ECD MS.

### 2.12 Analytical methods

Gas chromatography (GC-ECD, Agilent 7890 A, and USA) together with a 63Ni electron capture detector (ECD) and a packed column HP-5-5% phenyl methyl siloxan (30 m × 32 m i. d., film thickness 0.25 μm) was used to study the degradation of DDT. The process in the oven was as follows: The oven's temperature started at 75 °C and increased steadily over the next 25 min to 150 °C, where it was held for 0 min. After that, it increased by 5 min–280 °C, which it maintained for 10 min. There were 39 min throughout the run. The carrier gas utilized was nitrogen with a purity of 99.995 %, flowing at a rate of 60 mL per minute. Splitless mode was used to inject 2 μL from a 0.5-mL aliquot. Data analysis and instrument control were conducted using Chemstation software [86].

#### 2.12.1 Analysis of DDT, DDD and DDE

Gas chromatography (GC-ECD) combined with an electron capture detector (ECD) and a packed column of HP-5-5% phenyl methyl siloxan (30 m × 32 m i. d., film thickness 0.25 μm) was used to assess DDT residues. The oven settings were the same as previously reported [86]. Using Minitab 19 and ANOVA, percentages of the degradation data were evaluated. Using equation (2), the degradation efficiency of DDT and DDTr was determined [87].

(2) $$\%\;\text{DDT}\;\text{Break}\;\text{down}=\frac{\;{\;(\text{Peak}\;\text{area}\;}_{\text{DDE}}{+\text{Peak}\;\text{area}\;}_{\text{DDD}})\;}{\left({\text{Peak}\;\text{area}\;}_{\text{DDE}}{+\text{Peak}\;\text{area}\;}_{\text{DDD}\;}+\text{DDT}\right)}\times 100$$

### 2.13 Statistical analysis

Minitab version 2019 software (Minitab Inc., State College, PA, USA) was used to identify the mathematical modeling and optimal conditions of the DDT tolerance procedure. This program was utilized to produce the design matrix and examine the outcomes. Based on early studies, the upper and lower bounds for each independent variable were established. Using response surface methodology (RSM) based on Box-Behnken Design (BBD) and an ANOVA to identify significant variables, the amount of dry biomass of fungal mycelia in grams was assessed. The results were log-transformed (base 10) to get spores/mL. The media type, temperature, and pH were tested to see if they had a significant impact on the outcomes using a Fisher test. In order to validate the developed model, the probability (p-values) values were also measured; the p-value displayed is < 0.05, which is considered a significant model term. Multiple regression analysis techniques have been used to study the statistical significance. RSM was utilized to examine the importance of independent variables and their interactions, using Box-Behnken design analysis. A Pareto chart was also used to examine the standardized impacts of the independent factors and how they interacted with the dependent variables. The goodness-of-fit of the generated mathematical model to the experimental data was evaluated using a variety of descriptive and inferential statistical methods, including the p-value, F-test, R^2^ R^2^adj, sum of squares (SS), and mean sum of squares (MSS) tests [88]. Differences between means were considered statistically significant (p < 0.05) at a 5 % level of confidence [89,90]. The developed model is significant, as indicated by the model F-value of 2.13. The high F-value could be the result of noise. Furthermore, the lack of fit score (5.235) for the proposed model indicates that the lack of fit is not statistically significant. The model fits well, as evidenced by the model terms' non-significant lack of fit. The fungal sporulation response was precisely and consistently measured by the batch experiments, and the model's significance was further supported by the R (0.572). To determine how the factors and the response interact, the response surface model fits the experimental data [91].

## 3 Results

### 3.1 Identification of fungal isolate

**Fig. 2:**
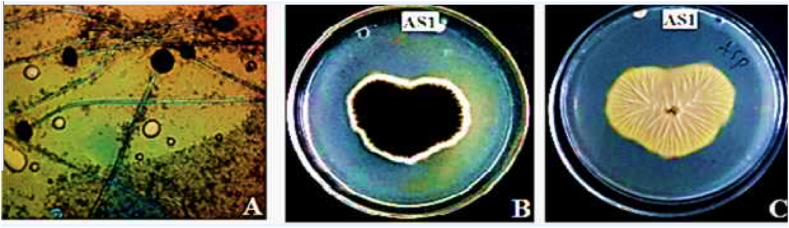
Cellular morphology of *A. niger* (A), Obverse side of *A. niger* (B)*,* Reverse side of *A. niger* (C).

**Fig. 3:**
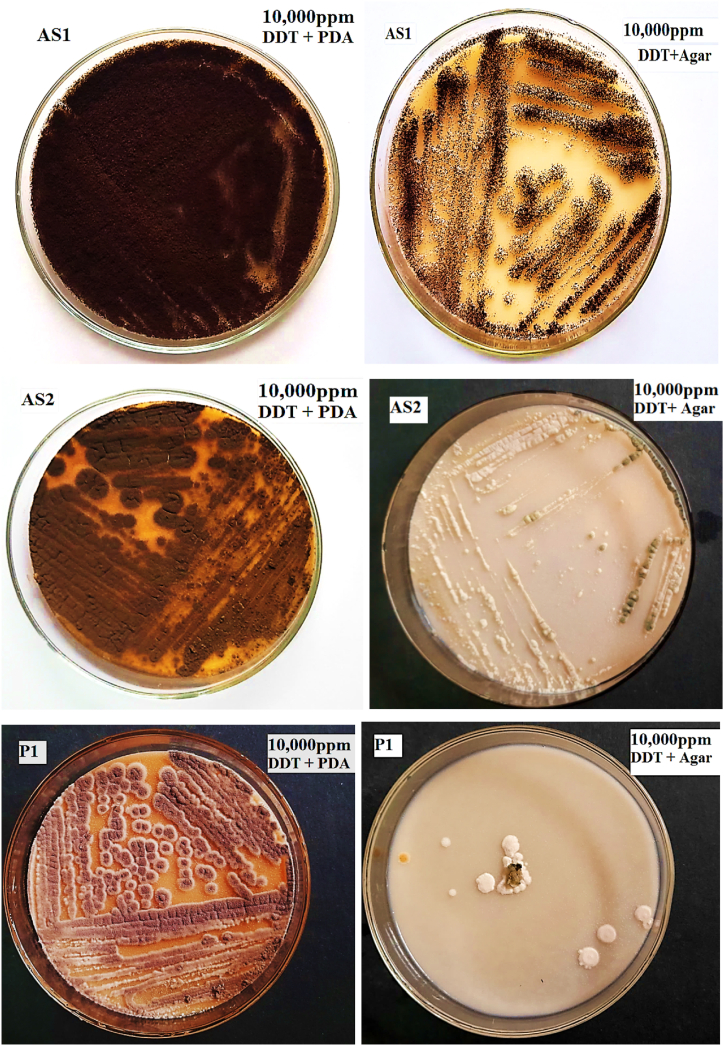
Qualitative fungal DDT-tolerant evaluation in 10,000ppmDDT amended media.

It takes an average of four days to identify the possible DDT-degrading fungi using morphological evaluation and conventional techniques. All isolates displayed distinct sizes, shapes, colors, and textures on PDA plates following a 3–7 day incubation period. The morphological traits of all selected filamentous fungi differed from one another. The Aspergillus species (spp.) grew rapidly and formed loose colonies on PDA plates, with all selected species exhibiting characteristic colors and shapes. For example, *A. niger* growths appeared whitish but turned black with time on PDA plates (Table 1). The microscopic examination with lactophenol cotton blue staining, hyaline septate hyphae, and biseriate phialides covering the entire vesicle with radiate conidial heads was observed (Table 1 and Fig. 2 (A-C)). The Penicillium spp. grew more slowly than the Aspergillus spp. On DDT amended and PDA medium, most Penicillium spp. produced radiated sulcata with a velvety colony surface, and some displayed colored exudates. It produced a soluble red pigment, which diffused into the medium, giving the colony a red or pink appearance (Fig. 3).

**Table 1:** Morphological characterization of isolate AS1.

**Isolate**	**Morphological characteristics of the colony**								
AS1	Surface colour		Margins		Reverse side		Elevations		Growth
	Dark brown to black		Entire		Without colour		Umbonate		Rapid
**Isolate**	**Microscopic characteristics**								
AS1	Hyphae	Conidiophore		Conidia		Phialides		Fruiting body	
	Branched	Length		Heads		Biseriate		Cleistothecia	
	Septate	200–400 μm		Blackish brown		Primary		Present	
		Diameter		Diameter		20 to 30 μ			
		7–10 μm		2.5–4 μm		Secondary			
		Vesicle		Ornametation		40 to 60 μ			
		Globose		Exine spiny		Globose

#### 3.1.1 Fungal identification by MALDI-TOF MS

Identification of filamentous fungus using the MALDI-TOF MS method took an average of 15 min (from the time the culture was made available for testing). Six fungal isolates were analyzed using MALDI-TOF MS technology. Two isolates (92.70 %) of these had genus-level identifications (identification scores ranging from 1.700 to 1.999) achieved. The last isolate to be chosen, AS1, was successfully identified as *A. niger* at the species level (identification scores of ≥2.000). Nevertheless, three strains (4.9 %; scores <1.700) were not detected by this approach. According to these findings, MALDI-TOF MS is a trustworthy method for identifying potentially DDT-degrading fungi because the method can quickly and accurately identify the majority of filamentous fungi.

### 3.2 Screening of DDT tolerance fungal isolates

Finally, three fungal isolates that could grow at a very high DDT dosage (10,000 ppm) ultimately screened for an optimization assay using DDT-amended solid media. They were given the designations AS1, AS2, and P1, as illustrated in Fig. 3. The growth of isolate AS1 is the highest as shown in Fig. 3 and will be used in the next DDT degradation experiment. Ultimately, after an extensive qualitative and quantitative assessment, isolate AS1 was identified as *A. niger* using MALDI-TOF mass spectrometry. It was proteomic analyses that allowed for the identification of *A. niger.* Optimization results revealed that *A. niger* had the maximum biomass and sporulation efficacy, as shown in Fig. 6, Fig. 7. The DDT-amended medium had higher tolerance index (TI) values than the control medium. The tolerance index (TI) values for the AS2 and P1 isolates were less than 100 %. However, the AS1 isolate had a TI value greater than 100 %, as demonstrated in Table 2. This finding demonstrated that, without exhibiting statistically significant differences (p > 0.05), fungal biomass production increased along with the established range of DDT concentration, temperature, and pH. Moreover, Table 2, Table 3 demonstrate a slight rise in sporulation.

**Table 2:** Fungal growth parameters for biomass production at different DDT concentrations, temperature and pH.

Fungal	Dry		DDT	Dry		Temp	Dry			Dry
Isolate	Biomass	TI	Conc.	Biomass	TI	(°C)	Biomass	TI	pH	Biomass
AS1	0.98 ± 0.22^a^	133	7000	0.87 ± 0.33^a^	118	35	0.81 ± 0.81^a^	110	10	0.87 ± 0.18^a^
P1	0.72 ± 0.24^b^	97	3500	0.81 ± 0.24^a^	110	30	0.81 ± 0.81^a^	110	7	0.79 ± 0.32^a^
AS2	0.72 ± 0.24^b^	97	0	0.74 ± 0.24^a^	100	25	0.79 ± 0.79^a^	107	4	0.77 ± 0.22^a^

The data represents the mean (±SD) of (n =3) and the same lowercase letters indicate a lack of statistically significant differences (p > 0.05) among means within the same column.

**Table 3:** Fungal growth parameters for spore production carried out at different DDT concentrations, temperature and pH.

Fungal Isolate	Log spore/mL	DDT conc. (ppm)	Log spore/mL	Temp. (°C)	Log spore/mL	pH	Log spore/mL
AS1	5.60 ± 0.32^a^	7000	5.51 ± 0.38^a^	35	5.56 ± 0.26^a^	10	5.52 ± 0.25^a^
P1	5.40 ± 0.32^ab^	0	5.48 ± 0.25^a^	25	5.43 ± 0.39^ab^	7	5.41 ± 0.29^a^
AS2	5.21 ± 0.31^b^	3500	5.30 ± 0.37^a^	30	5.30 ± 0.35^b^	4	5.26 ± 0.27^a^

The data represents the mean (±SD) of (n =3) and the same lowercase letters indicate a lack of statistically significant differences (p > 0.05) among means within the same column.

### 3.3 Screening significant variables using one way ANOVA statistical analysis

**Fig. 4:**
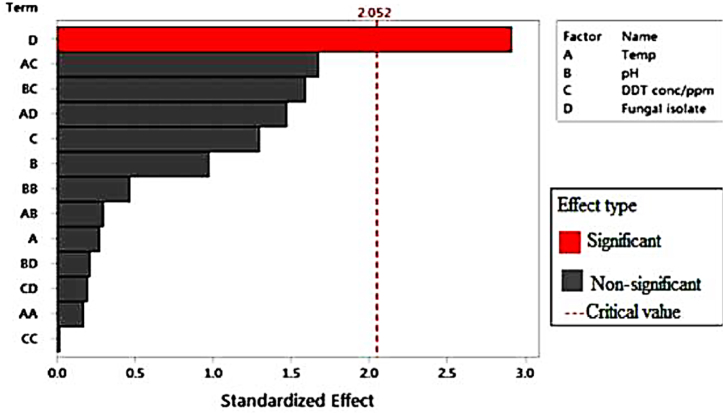
Pareto-regression graph indicating the effect of growth parameter on fungal biomass production.

**Fig. 5:**
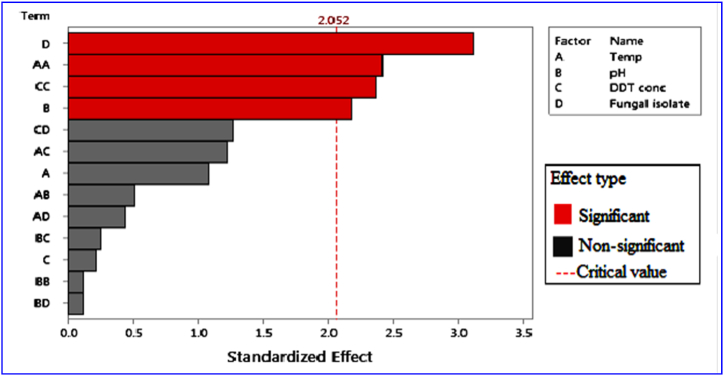
Pareto-regression graph indicating the effect of growth parameters on fungal spore production.

Fungal isolates were the only significant variable for the potential biomass production (p < 0.05), as indicated in Fig. 4. Temperature and fungal isolates were the most significant variables for potential sporulation efficacy (p < 0.05), as shown in Fig. 5. The incubation time remained fixed at ten days. As shown in Table 2, Table 3, an ANOVA data analysis also revealed that the pH and DDT concentrations had no effect on the potential production of fungal biomass or the ability to sporulate (p > 0.05).

**Fig. 6:**
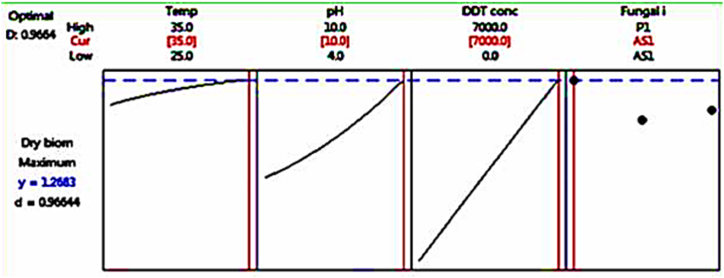
Optimization plot for biomass production.

**Fig. 7:**
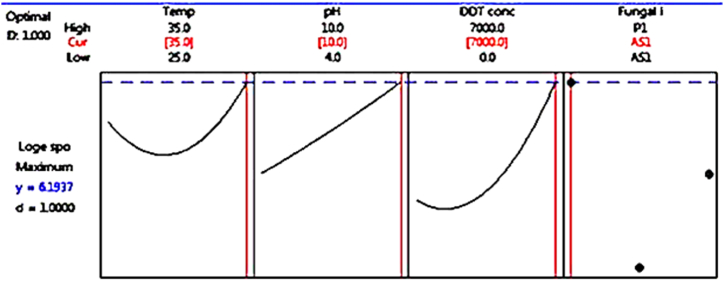
Optimization plot for spore production (log spore/ml).

### 3.4 Response surface methodology (RSM)-Based statistical optimization of biomass production and sporulation for the identification of significant factors

As seen in Fig. 6, Fig. 7, RSM is utilized to maximize biomass and sporulation capacity for the assessment of fungal DDT tolerance. The highest fungal mycelia dry biomass (0.981 g) and fungal spore production (5.6 log spores/mL) were found in fungal isolate AS1, according to optimization results. During the 10-day incubation period, this isolate grew well in PDB+7000 ppm DDT medium at a temperature of 35 °C and a pH value of 10, as seen in Fig. 6, Fig. 7.

#### 3.4.1 The main and interaction effect analysis between influencing factors

There was no significant difference detected between fungal isolates AS2 and P1 in fungal biomass production and sporulation efficacy (p > 0.05), as shown in Table 2, Table 3 The isolate AS1 produced significantly more biomass than the other two fungal isolates (p 0.05) (Table 2). Table 2, Table 3 show that there was no substantial variance (p > 0.05) in the development of fungal biomass and sporulation between the pH 4.0, 7.0, and 10 medium and the 1:1 ratio w/w of PDB DDT with 3500 and 7000 ppm DDT concentrations. The different temperature ranges (20, 25, and 35 °C) could not significantly influence the production of fungal dry biomass (p > 0.05), as shown in Table 2. However, the sporulation efficacy of isolate AS2 was significantly affected at 30 °C (p < 0.05), as shown in Table 3. As Fig. 4, Fig. 5 demonstrate, the two distinct factor interactions had no discernible effect on fungal biomass production and sporulation capability (p > 0.05). As illustrated in Fig. 5, the similar factor interactions (temperature vs. temperature) and (DDT concentration vs. DDT concentration) demonstrate that these variable interactions significantly influence fungal spore formation (p˂0.05).

### 3.5 Fungal DDT degradation assay by *A.* niger

**Fig. 8:**
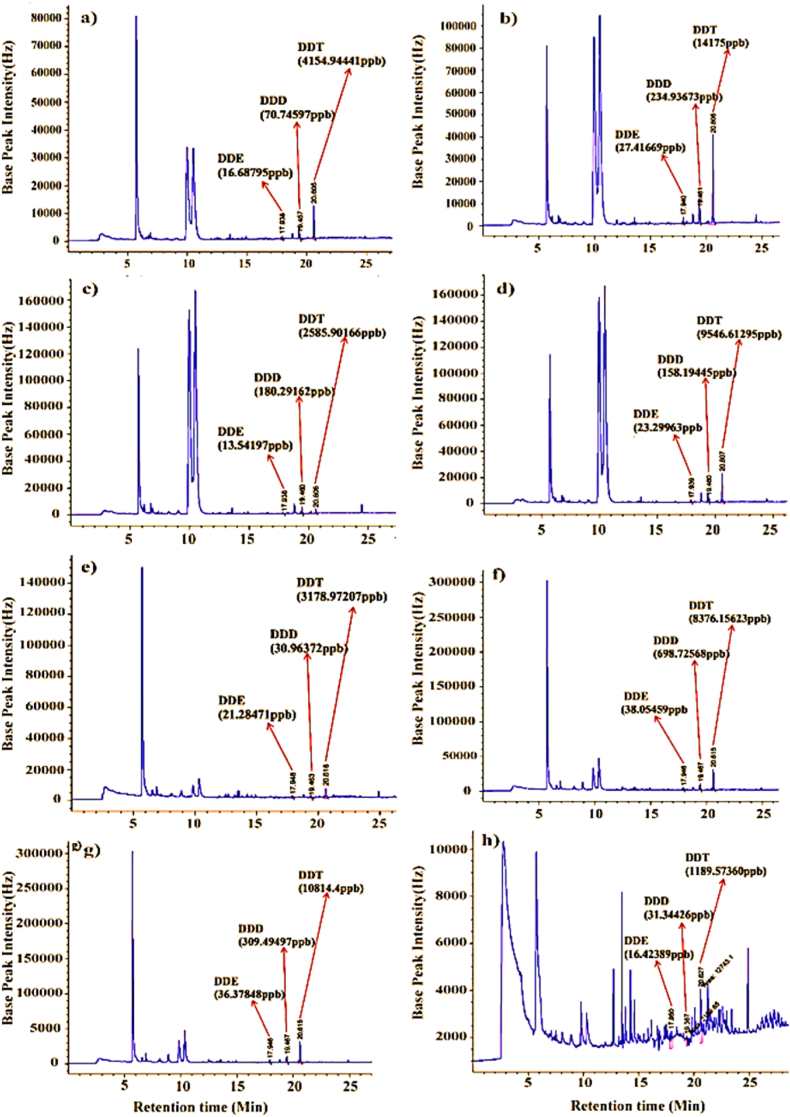
Gas chromatogram of DDT degradation by A. niger after 15 days cultured (supplied with 1750, 3500, 5250 and 7000ppmDDT): (a, b, c and d) electron capture of product extracted from 100 % pure DDT fungal mycelia and (e, f, g, and h) extracted from 1:1 ratio of PDB + DDT fungal mycelia respectively.

The amount of DDT degraded by *A. niger* was determined using GC-ECD technology. The constituents' retention times and electron capture are compared to reference samples. DDE and DDD were identified as metabolites of DDT breakdown by *A. niger* based on GC-ECD analysis Based on a comparison of retention times and mass spectra with known standard chemicals, DDE, DDD, and DDT are the metabolites that result from *A. niger's* breakdown of DDT as Fig. 8(a–h) demonstrates. It was determined that DDE was the peak at a retention time of 18.003 min. At 19.411 min, the peak's retention time was determined to be DDD. As its main metabolite, DDT was shown to be detected at a peak retention time of 20.666 min. The peak at a retention time of 20.666 min was identified as DDT, which was detected as its main metabolite. These results confirmed that DDT is not completely transformed into DDE and DDD, as Fig. 8 illustrates (a-h).

The only DDT levels found in 100 % supplemented with minimal salt media were 1.103 ppb, 0.896 ppb, 1.441 ppb, and 1.478 ppb. In addition, only 0.996 ppb, 1.260 ppb, 0.846 ppb, and 1.388 ppb were found in the 1:1 ratio of PDB and DDT media for mean percentage residual analysis, compared to the original concentration at 1750, 3500, 5250, and 7000 ppm, respectively. This GC-ECD result analysis confirmed that DDT has almost completely disappeared from both culture media by *A. niger*, as shown in Table 4 and Fig. 8 (a-h). *A. niger* has been studied as a very potential culture for biodegradation at such high DDT concentrations.

**Table 4:** Percentage residual and degradation analysis of DDT by *A.niger* in liquid MSM cultures during a 15-days inoculation detected by GC-ECD.

Initial DDT (ppm)	%Residual analysis			
	1750	3500	5250	7000
100 % DDT	1.103 ± 1.188^a^	0.896 ± 1.188^a^	1.441 ± 1.188^a^	1.478 ± 1.188^a^
Enriched	0.996 ± 1.188^a^	1.26 ± 1.188^a^	0.846 ± 1.188^a^	1.388 ± 1.188^a^
	%Degradation			
Initial DDT (ppm)	1750	3500	5250	7000
100 % DDT	98.89 ± 1.187^a^	99.104 ± 1.18^a^	98.559 ± 1.18^a^	98.522 ± 1.18^a^
Enriched	99.004 ± 1.18^a^	98.74 ± 1.18^a^	99.154 ± 1.18^a^	98.612 ± 1.18^a^

The data represents the (mean ± SE) (n =3), the same lowercase letters indicate a lack of statistically significant differences (p > 0.05) among means within the same row.

Very small amounts of DDT, DDD, and almost nothing at all of DDE were found in the extracts of all media cultures. As indicated in Table 5 and Fig. 8(a–h), the mean metabolic products for DDT, DDD, and DDE were calculated from the initial concentration of 1750, 3500, 5250, and 7000 ppm DDT. It was shaken for 15 days at 120 rpm and incubated at 27 ± 2 °C. These results clearly show that almost complete degradation of DDT by *A. niger* and its main metabolic products, DDD and DDE, has been investigated. The degradation of DDT is significantly different from that of DDD and DDE (p < 0.05), as shown in Table 5. This study could be the first case report for a fungal degradation of DDT up to 7000 parts per million in extremely high DDT concentrations by *A. niger*. According to Table 5, these findings verified that 96.636 %, 98.048 %, and 99.77 % of DDT, DDD, and DDE were broken down in liquid culture media. The results of this investigation also demonstrated *A. niger's* incredible ability to degrade DDT at high concentrations

**Table 5:** Percentage residual and degradation analysis of DDT and its metabolic products by *A.niger* in liquid MSM culture during15 a day's incubation period.

Metabolic products	DDT	DDD	DDE
(%) Residual	3.358 ± 1.92^a^	1.952 ± 1.92^ab^	0.23 ± 1.92^b^
% degradation	96.636 ± 1.92^b^	98.048 ± 1.92^ab^	99.77 ± 1.92^a^

The data represents the (mean ± SE) (n =3), and the same lowercase letters indicate a lack of statistically significant differences (p > 0.05) among means within the same row.

The results of this research study demonstrated that elevating initial DDT concentrations from 1750 ppm up to 7000 ppm in both DDT amended media (PDB-enriched DDT and 100 % pure DDT) had no discernible effects on the effectiveness of fungal degradation of DDT by *A. niger* (p > 0.05), as shown in Table 4. Additionally, DDT and its metabolic product, DDE, nearly disappeared, according to the results of the GC-ECD analysis. Nevertheless, Table 5 shows that there was no discernible difference between DDT and its metabolic product, DDD (p > 0.05). This investigation also demonstrated the screened fungal isolate *A. niger's v*ery strong DDT-degrading capacity, which breaks down DDT and its metabolic products effectively.

## 4 Discussions

One of the most innovative and economical options for cleaning up and eliminating DDT from the environment is biodegradation. This DDT degradation mechanism allows microorganisms to digest contaminants. It is only effective in conditions that encourage microbial growth and activity. According to Boelan et al. [92], *G. lucidum* Chaaim-001 BCU thrived poorly at temperatures over 30 °C but well at 30 °C. On the other hand, the present *A. niger* was able to thrive in a broad range of environmental circumstances. It also has a higher tolerance to elevated DDT exposures up to 7000 ppm. Upadhyay et al.'s [93] study found that under optimal physiochemical conditions, the main mechanism of DDT breakdown by microorganisms was identified. The impact of additional nutrient sources, different growing conditions, temperature, pH, and time intervals on POP biodegradation was examined in the recent study by Barrios San Martín [52]. The microbes that might breakdown DDT at high concentrations previously documented in the literature are not the same as the current *A. niger*. It may use DDT as its only carbon source. It is highly tolerant of high DDT concentrations, alkaline and acidic media, and a broad temperature range. This demonstrates *A. niger's* adaptability to a variety of environments [94]. Research by Govarthanan et al. [84] found that certain fungi can break down POPs, including DDT. An effective biodegradation system needs a competent microbes that capable of transforming toxic DDT pollutants into nontoxic ones. Very efficient microbes could use the DDT contaminant as a source of carbon and energy for growth and metabolism. A study by Boelan et al. [92] found that after seven days of incubation, *P. aeruginosa* was able to break down 90 % of DDT in a 7-mL volume of bacteria. The current isolate, *A. niger*, has the potential to degrade DDT and its metabolites (DDE and DDD) by 98%–99.77 % at 10^8^ spores/mL within a 15-day incubation period. According to a study conducted by Maqboo et al. [95], *A. niger* completely mineralized endosulfan pesticide (300 mg/L) within 12 days of incubation. Ebsa et al. [60] also studied the co-inoculated mixed culture of T. koningii and *A. niger* that degraded almost entirely DDT and its primary metabolites, DDE and DDD (98–99.99 %) at 10^8^ spores/mL in very high DDT concentrations up to 7000 ppm within a 15-day incubation period. In this study DDT is the only carbon source used by *A. niger* potentially. The GC-ECD results revealed that *A. niger* can degrade DDT efficiently up to 7000 ppm within 15 days of incubation.

According to Ceci et al.'s study [59], approximately 99 % of the 150 mg/L DDT concentration has been eliminated. Álvarez et al.'s study [43] additionally investigated the marine bacterium *Paracoccus* sp. DDT-21, which quickly detoxified DDT at a concentration of 50 mg/L. Recent research, however, has demonstrated that *A. niger* is not the same as the previously documented microorganisms. It was discovered that 98–99 % of DDT disappeared from the DDT-enriched PDB culture medium and 100 % pure DDT supplemented with minimal salt medium when *A. niger* was grown in liquid MSM supplemented with a very high concentration of DDT (1750–7000 ppm) and incubated at a temperature of 27 ± 2 °C for 15 days on a shaker. To the best of our knowledge and recently reviewed literature, there hasn't been any information reported on the ability of Aspergillus species to degrade DDT at very high concentrations, up to 7000 ppm. Therefore, this study confirmed the capability of *A. niger* to degrade DDT in liquid MSM with high DDT concentrations efficiently. Although this isolate has a unique trait for DDT degradation capability, it needs rigorous study to determine if it is accompanied by genetic factors or not since it was isolated from a polluted tropical environment surrounding industries. This study's findings showed that the dominant degradation was the transformation of DDT to DDE, which revealed that 99.77 % disappeared from this very high DDT concentration liquid culture medium. GC-ECD analysis illustrated three metabolic products of DDT degradation by *A. niger*. This result is similar to a study conducted by Purnomo et al. [96] showing that a strain of brown rot fungi produced DDE, DDD, and DBP from DDT degradation. In the liquid cultures, there was no DBP found in the nutrient solutions. The DDT concentrations in liquid cultures were higher than DDE and DDD, suggesting that DDT is the main metabolic product of DDT degradation by *A. niger*, which is similar to the research of Bumpus and Aust [94]. on DDT degradation by white rot fungi and P. chrysosporium.

In comparison, numerous current studies have reported that the degradation of DDT by co-inoculated cultures of *A. niger* and T. koningii is more effective than that of *P. aeruginosa*. The hybrid cultures of *T. koningii* and *A. niger*, and *P. aeruginosa* degraded DDT by approximately 99 % and 90 %, respectively [60,95]. This may be due to the fact that mixed cultures of *A. niger* and *T. koningii* have the capacity to produce lots of genes through an interaction effect. It could encompass both the sequential degradation process and *P. aeruginosa's* capacity to generate a rhamnolipid biosurfactant, which could potentially improve the solubility of DDT.

**Fig. 9:**
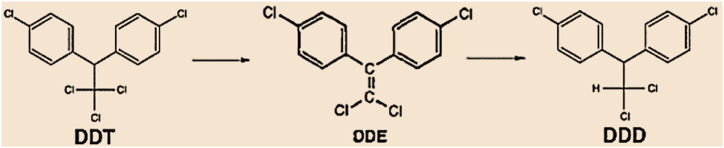
The proposed pathway for the myco-degradation of DDT by *A. niger*.

While *P. aeruginosa* generated DDD and DDMU, the *A. niger* and *T. koningii's* combination cultures*,* as well as *A. niger*, solely metabolized DDT to DDE and DDD, based on the metabolic products' identification by GC-MS and GC-ECD MS. This result indicated that *A. niger* has an important role in DDT transformation. *A. niger* transforms DDT to DDE via dehydrochlorination, followed by DDE reductive dechlorination. In this study, the proposed DDT degradation pathway by *A. niger* is shown in Fig. 9. The brown rot fungal degradation process, which could convert DDT to DDE as a metabolic product by removing chloride ions and then further transform to DDD by reductive dechlorination, was similar to this DDT degradation pathway [96]. According to a study reported by Purnomo et al. [97], this DDT degradation pathway is also different from the proposed pathway conducted by fungal-bacterial co-cultures of *P. eryngii* and *R. pickettii*, which transform DDT to DDD via reductive dechlorination, followed by dehydrochlorination to DDE. DDE then underwent dechlorination to DDMU, thereby showing that DDMU was subjected to further transformation by *P. eryngii* and that DDD was transformed by co-cultures. Schuster et al. [98] studied the toxicity of *A. niger* and found that it should be handled wisely to avoid the production of spore dust, like all other filamentous fungi. But in terms of allergies or mycopathology, it is not very problematic when compared to other filamentous fungi.

## 5 Conclusion

DDT and its residues are persistent in the environment, contaminating areas, animals, and human health. The current study attempted to isolate various fungi capable of decomposing DDT from samples of agricultural soil and agro-industrial waste water effluent. Out of 40 potential DDT-tolerant fungi, three fungal isolates were chosen for the optimization process. We examined and improved four different growth factors: pH, temperature, DDT concentration, and fungal isolate. According to the optimization results, after 10 days of incubation at 35 °C and pH 10, *A. niger* had the highest biomass and spore count in DDT amended liquid media. The ability of fungal biomass production to sporulate or grow is unaffected by the high amounts of DDT (3500 and 7000 ppm). After 15 days of incubation at 27 ± 2 °C on a shaker at 120 rpm, the highest degradation analysis (∼99.77 %) was found at DDE, and the lowest degradation analysis (∼96.6 %) was found at DDT using GC-ECD. *A. niger* is therefore a viable option for the elimination and/or detoxification of DDT and its breakdown products from contaminated areas.

Fungal additions that may enhance bioavailability and break down DDT in contaminated environments have been the subject of recent batch research. Under concentrated DDT-amended liquid media conditions, *A. niger's* capacity for DDT degradation contributes more effectively to the bioaugmentation process. Even though DDT is one of the most difficult pollutants to break down, recent advances in in vitro research have opened up new avenues for *A. nige*r to almost completely break down DDT and its primary residues, DDD and DDE, up to 7000 ppm DDT concentration. Using solely *A. niger*, no investigation has been able to shed light on microbial DDT breakdown with such a high concentration of DDT. This species is also adaptable enough to thrive in a variety of challenging and favorable environmental settings.

Lastly, the myco-remediation of DDT by *A. niger* in batch investigations is the main emphasis of the current study. Nonetheless, one of the primary difficulties is keeping the *A. niger* stable and operational for an extended period of time. Another issue is operating in environmentally relevant, but non-axenic, settings. There are extra difficulties when there are co-existing environmental contaminants, including azo dye, heavy metals, and HAP. Furthermore, altering the ambient circumstances should regulate these parameters that influence microbial development patterns. Researching continuous flow technologies is crucial for large-scale industrial applications. Further research is required to fully mineralize DDT in actual environmental contexts. This research includes an environmental microbial metagenomics study and a fungal tolerance study against a variety of environmental toxins, such as heavy metals, azo dye, PAHs, and others. This study has to look into whether or how developed fungal cultures outcompete natural microorganisms in environmental matrices and how non-axenic conditions affect them. It is crucial to carry out field applications at various spots. While transgenic microbes have made significant progress in detoxifying and breaking down DDT contaminants, more study is needed to optimize their environmental survivability. In order to promote the growth of microorganisms more successfully and efficiently. It is essential to conduct a thorough analysis of the environmental factors, DDT degradation operating parameters, and DDT bioremediation process mechanisms. It is necessary to develop enzyme systems that produce the best yields and efficiency with the least amount of energy, water, and nutrients. Despite the fact that genetic engineering techniques produce more enzymes more effectively, there is still a budgetary barrier that needs to be addressed.

## Data availability statement

Data included in article/supp. material/referenced in article.

## Additional information

No additional information is available for this paper.

## CRediT authorship contribution statement

**Girma Ebsa:** Writing – original draft, Methodology, Investigation, Formal analysis, Data curation, Conceptualization. **Birhanu Gizaw:** Writing – review & editing, Validation, Supervision, Methodology, Investigation. **Mesele Admassie:** Writing – review & editing, Visualization, Supervision, Software, Data curation. **Asnake Desalegn:** Writing – review & editing, Visualization, Supervision, Methodology. **Tesfaye Alemu:** Writing – review & editing, Validation, Supervision, Resources, Project administration, Methodology, Conceptualization.

## Declaration of competing interest

The authors declared that they don't have any known competing ﬁnancial interests or personal relationships that could have appeared to inﬂuence the work reported in this paper.

## References

[1] Puri M., Gandhi K., Kumar M.S. (2023). Emerging environmental contaminants: a global perspective on policies and regulations. J. Environ. Manag..

[2] Gudi S., Alagappan P., Raigar O.P., Halladakeri P., Gowda R.S., Kumar P., Singh G., Tamta M., Susmitha P., Amandeep D.K., Saini (2024). Fashion meets science: how advanced breeding approaches could revolutionize the textile industry. Crit. Rev. Biotechnol..

[3] Kaur R., Kaur H., Singh S., Jagota N., Sharma A., Sharma A. (2024). Organic Micropollutants in Aquatic and Terrestrial Environments.

[4] Chaudhari Y.S., Kumar P., Soni S., Gacem A., Kumar V., Singh S., Yadav V.K., Dawane V., Piplode S., Jeon B.H., Ibrahium H.A. (2023). An inclusive outlook on the fate and persistence of pesticides in the environment and integrated eco-technologies for their degradation. Toxicol. Appl. Pharmacol..

[5] Kumari S., Das S. (2023). Bacterial enzymatic degradation of recalcitrant organic pollutants: catabolic pathways and genetic regulations. Environ. Sci. Pollut. Control Ser..

[6] Wang X., Oba B.T., Wang H., Luo Q., Liu J., Tang L., Yang M., Wu H.L. (2023). Sun, Degradation of DDT by a novel bacterium, Arthrobacter globiformis DC-1: efficacy, mechanism and comparative advantage. Water.

[7] Burgos-Aceves M.A., Migliaccio V., Di Gregorio I., Paolella G., Lepretti M., Faggio C., Lionetti L. (2021). 1, 1, 1-trichloro-2, 2-bis (p-chlorophenyl)-ethane (DDT) and 1, 1-Dichloro-2, 2-bis (p, p’-chlorophenyl) ethylene (DDE) as endocrine disruptors in human and wildlife: a possible implication of mitochondria. Environ. Toxicol. Pharmacol..

[8] Kolan A.S., Hall J.M. (2023). Association of preterm birth and exposure to endocrine disrupting chemicals. Int. J. Mol. Sci..

[9] Padayachee K., Reynolds C., Mateo R., Amar A. (2023). A global review of the temporal and spatial patterns of DDT and dieldrin monitoring in raptors. Sci. Total Environ..

[10] Vinuales J.E., Bartels L., Paddeu F. (2016). Exceptions in International Law.

[11] Bohlin-Nizzetto P., Aas W., Halvorsen H.L., Nikiforov V., Pfaffhuber K.A. (2021).

[12] Mekonen S., Ibrahim M., Astatkie H., Abreha A. (2021). Exposure to organochlorine pesticides as a predictor to breast cancer: a case-control study among Ethiopian women. PLoS One.

[13] Olani A.B. (2024). Neither laissez-faire nor prohibition: the khat regulation policy preferences of people who chew khat and local social service providers in Ethiopia. Drugs Educ. Prev. Pol..

[14] Siraj J., Ejeta F. (2024). Analysis of pesticide residues in fruits and vegetables using gas chromatography-mass spectrometry: a case from West Omo and Bench-Sheko Zone, Southwest Ethiopia. Int. J. Environ. Anal. Chem..

[15] Bhatt P., Verma A., Gangola S., Bhandari G., Chen S. (2021). Microbial glycoconjugates in organic pollutant bioremediation: recent advances and applications. Microb. Cell Factories.

[16] Goel S.C. (1986).

[17] Tang C., Chen Z., Huang Y., Solyanikova I.P., Mohan S.V., Chen H., Wu Y. (2023). Occurrence and potential harms of organochlorine pesticides (OCPs) in environment and their removal by periphyton. Crit. Rev. Environ. Sci. Technol..

[18] Ahmed T., MiShr P., Singh A.B., TyAgi U., Banerjee B.D. (2022). Pesticides and human health: antioxidants and heat shock proteins as modulators of cell survival signal. J. Clin. Diagn. Res..

[19] Keswani C., Dilnashin H., Birla H., Roy P., Tyagi R.K., Singh D., Rajput V.D., Minkina T., Singh S.P. (2022). Global footprints of organochlorine pesticides: a pan-global survey. Environ. Geochem. Health.

[20] Zhang Y., Gao Y., Liu Q.S., Zhou Q., Jiang G. (2024). Chemical contaminants in blood and their implications in chronic diseases. J. Hazard Mater..

[21] Liang Z., Abdelshafy A.M., Luo Z., Belwal T., Lin X., Xu Y., Wang L., Yang M., Qi M., Dong Y., Li L. (2022). Occurrence, detection, and dissipation of pesticide residue in plant-derived foodstuff: a state-of-the-art review. Food Chem..

[22] Ansari F., Lee C.C., Rashidimehr A., Eskandari S., Ashaolu T.J., Mirzakhani E., Pourjafar H., Jafari S.M. (2023). The role of probiotics in improving food safety; detoxification of heavy metals and chemicals. Toxin Rev..

[23] Wu M., Miao J., Zhang W., Wang Q., Sun C., Wang L., Pan L. (2024). Occurrence, distribution, and health risk assessment of pyrethroid and neonicotinoid insecticides in aquatic products of China. Sci. Total Environ..

[24] Mehta R.V., Sreenivasa M.A., Mathew M., Girard A.W., Taneja S., Ranjan S., Ramakrishnan U., Martorell R., Ryan P.B., Young M.F. (2020). A mixed-methods study of pesticide exposures in Breastmilk and Community & Lactating Women's perspectives from Haryana, India. BMC Publ. Health.

[25] Malusá E., Tartanus M., Danelski W., Miszczak A., Szustakowska E., Kicińska J., Furmanczyk E.M. (2020). Monitoring of DDT in agricultural soils under organic farming in Poland and the risk of crop contamination. Environ. Manag..

[26] Li B.A., Li B.M., Bao Z., Li Q., Xing M., Li B. (2023). Dichlorodiphenyltrichloroethane for malaria and agricultural uses and its impacts on human health. Bull. Environ. Contam. Toxicol..

[27] Alemayehu W. (2001). Response to PAN UK Questionnaire for African Government Regulators.

[28] Haylamicheal I.D., Dalvie M.A. (2009). Disposal of obsolete pesticides, the case of Ethiopia. Environ. Int..

[29] Karlsson S. (2006).

[30] Ayele S., Mamo Y., Deribe E., Eklo O.M. (2022). Organochlorine pesticides and polychlorinated biphenyls in carnivorous waterbird species from Lake Ziway, Ethiopia. SN Appl. Sci..

[31] Mekonen S., Ambelu A., Wondafrash M., Kolsteren P., Spanoghe P. (2021). Exposure of infants to organochlorine pesticides from breast milk consumption in southwestern Ethiopia. Sci. Rep..

[32] Kuang L., Hou Y., Huang F., Hong H., Sun H., Deng W., Lin H. (2020). Pesticide residues in breast milk and the associated risk assessment: a review focused on China. Sci. Total Environ..

[33] Atnafie S.A., Muluneh N.Y., Getahun K.A., Woredekal A.T., Kahaliw W. (2021). Research article pesticide residue analysis of khat leaves and health risks among khat chewers in the amhara region, northwestern Ethiopia. Journal of Environmental and Public Health.

[34] Asadikaram G., Pourghadamyari H., Abolhassani M., Abbasi-Jorjandi M., Faramarz S., Yousefi F., Salimi F., Malekpour Afshar R., Asadikaram P., Shafiepour M. (2024). Oxidative stress induction by OCPs and OPPs pesticides may cause lung cancer incidence. Toxin Rev..

[35] K.O.Y. Habibullah, *Biodegradation Of Organochlorine Insecticide DDT* (Doctoral Dissertation, Tohoku University).

[36] Ngu W.J., Hua A.K., Zakaria Z., Jusoh M.N.H. (2024). Review on organochlorine pollution in Malaysia. Sustainable Environmental Insight.

[37] Bokade P., Purohit H.J., Bajaj A. (2021). Myco-remediation of chlorinated pesticides: insights into fungal metabolic system. Indian J. Microbiol..

[38] Prathima G. (2023). Environmental bioremediation of DDT-contaminated soil by using the mushroom extract of L. Edodes. Curr. Trends Biotechnol. Pharm..

[39] Balawejder M., Antos P., Czyjt-Kuryło S., Józefczyk R., Pieniążek M. (2014). A novel method for degradation of DDT in contaminated soil. Ozone: Sci. Eng..

[40] Z. Yilmaz Serçinoğlu, Multiobjective Optimization of Lignocellulolytic Enzyme Production by the White-Rot Fungus Pycnoporus Sanguineus (DSMZ 3024) Using Hazelnut Husk as a Substrate.

[41] Kovačić Đ., Lončarić Z., Jović J., Samac D., Popović B., Tišma M. (2022). Digestate management and processing practices: a review. Appl. Sci..

[42] Tarla D.N., Erickson L.E., Hettiarachchi G.M., Amadi S.I., Galkaduwa M., Davis L.C., Nurzhanova A., Pidlisnyuk V. (2020). Phytoremediation and bioremediation of pesticide-contaminated soil. Appl. Sci..

[43] Álvarez S.P., Ardisana E.F., Morales S.G., Monarez A.P. (2021). Rhizobiont in Bioremediation of Hazardous Waste.

[44] Sharma S., Saxena S., Mudgil B., Vats S. (2022). Biological Approaches to Controlling Pollutants.

[45] Kumar V., Pallavi P., Sen S.K., Raut S. (2024). Harnessing the potential of white rot fungi and ligninolytic enzymes for efficient textile dye degradation: a comprehensive review. Water Environ. Res..

[46] Al-Rashed S., Marraiki N., Syed A., Elgorban A.M., Prasad K.S., Shivamallu C., Bahkali A.H. (2021). Bioremediation characteristics, influencing factors of dichlorodiphenyltrichloroethane (DDT) removal by using non-indigenous Paracoccus sp. Chemosphere.

[47] Reddy K., Jose S., Fayaz T., Renuka N., Ratha S.K., Kumari S., Bux F. (2024). Microbe-Assisted bioremediation of pesticides from contaminated habitats. Bioremediation for Sustainable Environmental Cleanup.

[48] Baite N.A., Saikia N., Yadav N., Bhutia D.D. (2024). Microbiome-Assisted Bioremediation.

[49] Barrios San Martín Y. (2011). Biorremediación: una herramienta para el saneamiento de ecosistemas Marinos contaminados con petróleo. Biotecnol. Apl..

[50] Vashishth A., Tehri N., Kumar P. (2019). The potential of naturally occurring bacteria for the bioremediation of toxic metals pollution. Brazilian Journal of Biological Sciences.

[51] Azuazu I.N., Sam K., Campo P., Coulon F. (2023). Challenges and opportunities for low-carbon remediation in the Niger Delta: towards sustainable environmental management. Sci. Total Environ..

[52] Bakar N.A., Karsani S.A., Alias S.A. (2020). Fungal survival under temperature stress: a proteomic perspective. PeerJ.

[53] Kumar V., Sarma V.V., Thambugala K.M., Huang J.J., Li X.Y., Hao G.F. (2021). Ecology and evolution of marine fungi with their adaptation to climate change. Front. Microbiol..

[54] Vaksmaa A., Guerrero-Cruz S., Ghosh P., Zeghal E., Hernando-Morales V., Niemann H. (2023). Role of fungi in bioremediation of emerging pollutants. Front. Mar. Sci..

[55] Sanhueza T., Hernández I., Sagredo-Sáez C., Villanueva-Guerrero A., Alvarado R., Mujica M.I., Fuentes-Quiroz A., Menendez E., Jorquera-Fontena E., Valadares R.B.D.S., Herrera H. (2024). Juvenile plant–microbe interactions modulate the adaptation and response of forest seedlings to rapid climate change. Plants.

[56] Velasco A., Aburto-Medina A., Ortíz I. (2020). Enhancement of the DDT reductive dehalogenation by different cosubstrates: role of sulfidogenic and biogeochemical processes in soil. Appl. Geochem..

[57] Xu H.J., Bai J., Li W., Murrell J.C., Zhang Y., Wang J., Luo C., Li Y. (2021). Mechanisms of the enhanced DDT removal from soils by earthworms: identification of DDT degraders in drilosphere and non-drilosphere matrices. J. Hazard Mater..

[58] Jones A.D., MoreheadJr A.T., Yang Y. (2023). Degradation and extraction of organochlorine pollutants from environmental solids under subcritical water conditions. Molecules.

[59] Ceci A., Pinzari F., Russo F., Persiani A.M., Gadd G.M. (2019). Roles of saprotrophic fungi in biodegradation or transformation of organic and inorganic pollutants in co-contaminated sites. Appl. Microbiol. Biotechnol..

[60] Ebsa G., Gizaw B., Alemu T. (2024). Screening, characterization and optimization for synergistic interaction of potential dichlorodiphenyltrichloroethane degrading fungi isolated from agro-industrial effluent and farm soil. Biocatal. Agric. Biotechnol..

[61] Ebsa G., Gizaw B., Admassie M., Degu T., Alemu T. (2024). The role and mechanisms of microbes in dichlorodiphenyltrichloroethane (DDT) and its residues bioremediation. Biotechnology Reports.

[62] Czaplicki L.M., Cooper E., Ferguson P.L., Stapleton H.M., Vilgalys R., Gunsch C.K. (2016). A new perspective on sustainable soil remediation—case study suggests novel fungal genera could facilitate in situ biodegradation of hazardous contaminants. Remed. J..

[63] Akpasi S.O., Anekwe I.M.S., Tetteh E.K., Amune U.O., Shoyiga H.O., Mahlangu T.P., Kiambi S.L. (2023). Mycoremediation as a potentially promising technology: current status and prospects—a review. Appl. Sci..

[64] Mohamed H.I., Aal M.H.A., El-Mahdy O.M. (2024). Fungal Secondary Metabolites.

[65] Santacruz G., Bandala E.R., Torres L.G. (2005). Chlorinated pesticides (2, 4-D and DDT) biodegradation at high concentrations using immobilized *Pseudomonas fluorescens*. Journal of Environmental Science and Health Part B.

[66] Okçu G.D., Pakdil N.B., Ökten H.E., Yalçuk A. (2018). A Box-Behnken design (BBD) optimization of the photocatalytic degradation of 2, 4-dichlorophenoxyacetic acid (2, 4-D) using TiO2/H2O. Desalination Water Treat..

[67] Okçu G.D. (2022). Optimization of hybrid sonophotocatalytic decolorization of rhodamine B (RhB) dye using TiO2 nanocatalyst. Düzce Üniversitesi Bilim ve Teknoloji Dergisi.

[68] Nabilah B., Purnomo A.S., Prasetyoko D., Rohmah A.A. (2023). Methylene Blue biodecolorization and biodegradation by immobilized mixed cultures of Trichoderma viride and Ralstonia pickettii into SA-PVA-Bentonite matrix. Arab. J. Chem..

[69] Geethamani R., Soundara B., Kanmani S. (2023). Emerging contaminants in the environment and bioremediation control strategies–A review. IOP Conf. Ser. Earth Environ. Sci..

[70] OECD (2019).

[71] Suman S. (2021). Tanuja, isolation and characterization of a bacterial strain *Enterobacter cloacae* (accession No. KX438060. 1) capable of degrading DDTs under aerobic conditions and its use in bioremediation of contaminated soil. Microbiol. Insights.

[72] Nadeau L.J., Menn F.M., Breen A., Sayler G.S. (1994). Aerobic degradation of 1, 1, 1-trichloro-2, 2-bis (4-chlorophenyl) ethane (DDT) by *Alcaligenes eutrophus* A5. Appl. Environ. Microbiol..

[73] Persiani A.M., Maggi O., Montalvo J., Casado M.A., Pineda F.D. (2008). Mediterranean grass- land soil fungi: patterns of biodiversity, functional redundancy and soil carbon storage. Plant Biosyst..

[74] Bedine M.A.B., Taïeb N., Agriopoulou S., Miché L., Moussango D., Sameza M.L., Dupuy N., Roussos S., Fekam Boyom F. (2022). Isolates and Analysis of Their Antagonist Traits against *Lasiodiplodia*.

[75] Susila E., Maulina F., Emilda D. (2023). Characterization and identification of Trichoderma on shallots isolated from three elevation regions in West Sumatra, Indonesia. BIODIVERSITAS.

[76] Silva A.M., Jones D.L., Chadwick D.R., Qi X., Cotta S.R., Araújo V.L., Matteoli F.P., V G., Lacerda-Júnior G.V., Pereira A.P., Fernandes-Júnior P.I., Cardoso E.J. (2023). Can arbuscular mycorrhizal fungi and rhizobacteria facilitate 33P uptake in maize plants under water stress?. Microbiol. Res..

[77] Watanabe T. (2010).

[78] Huang D., Gao L., Zhu S., Qiao L., Liu Y., Ai Q., Xu C., Wang W., Lu M., Zheng M. (2023). Target and non-target analysis of organochlorine pesticides and their transformation products in an agrochemical-contaminated area. Chemosphere.

[79] Hleba L., Hlebova M., Kovacik A., Petrova J., Maskova Z., Cubon J., Massanyi P. (2022). Use of MALDI-TOF MS to discriminate between aflatoxin B1-producing and non-producing strains of *Aspergillus flavus*. Molecules.

[80] Sanguinetti M., Posteraro B. (2017). Identification of molds by matrix-assisted laser desorption ionization–time of flight mass spectrometry. J. Clin. Microbiol..

[81] Ceci A., Spinelli V., Massimi L., Canepari S., Persiani A.M. (2020). Fungi and arsenic: tolerance and bioaccumulation by soil saprotrophic species. Appl. Sci..

[82] Russo F., Ceci A., Pinzari F., Siciliano A., Guida M., Malusà E., Tartanus M., Miszczak A., Maggi O., Persiani A.M. (2019). Bioremediation of dichlorodiphenyltrichloroethane (DDT)-contaminated agricultural soils: potential of two autochthonous saprotrophic fungal strains. Appl. Environ. Microbiol..

[83] Mikolasch A., Reinhard A., Alimbetova A., Omirbekova A., Pasler L., Schumann P., Kabisch J., Mukasheva T., Schauer F. (2016). From oil spills to barley growth–oil‐degrading soil bacteria and their promoting effects. J. Basic Microbiol..

[84] Govarthanan M., Manikandan S., Subbaiya R., Krishnan R.Y., Srinivasan S., Karmegam N., Kim W. (2022). Emerging trends and nanotechnology advances for sustainable biogas production from lignocellulosic waste biomass: a critical review. Fuel.

[85] Wilkes T.I., Warner D.J., Edmonds-Brown V., Davies K.G., Denholm I. (2021). The tripartite Rhizobacteria-AM fungal-host plant relationship in winter wheat: impact of multi-species inoculation, tillage regime and naturally occurring Rhizobacteria species. Plants.

[86] Zhou E., Li F., Zhang D., Li D., Xu Z., Jia R., Jin Y., Song H., Li H., Wang Q., Wang J. (2022). Direct microbial electron uptake as a mechanism for stainless steel corrosion in aerobic environments. Water Res..

[87] Ma J., Pan L.B., Yang X.Y., Liu X.L., Tao S.Y., Zhao L., Qin X.P., Sun Z.J., Hou H., Zhou Y.Z. (2016). DDT, DDD, and DDE in soil of Xiangfen County, China: residues, sources, spatial distribution, and health risks. Chemosphere.

[88] Aziz N.A.M., Yunus R., Rashid U., Syam A.M. (2014). Application of response surface methodology (RSM) for optimizing the palm-based pentaerythritol ester synthesis *industrial Crops and products*.

[89] Purnomo A.S., Putra S.R., Shimizu K., Kondo R. (2014). Biodegradation of heptachlor and heptachlor epoxide-contaminated soils by white-rot fungal inocula. Environ. Sci. Pollut. Control Ser..

[90] Purnomo A.S., Sariwati A., Fatmawati S., Puspitasari F.E. (2023). Effect of the coconut coir (cocos nucifera) as a growth medium for *Pleurotus ostreatus* (oyster mushroom) on mineral and vitamin B contents. HAYATI Journal of Biosciences.

[91] Mani V.M., Soundari A.P.G., Salin K.P., Mohankumar R., Preethi K., Al Obaid S., Alharbi S.A., Jhanani G.K., Shanmugam S. (2023). Optimization parameters for the production of dimer of epicatechin from an endophytic fungus *Curvularia australiensis* FC2AP using response surface methodology (RSM). Environ. Res..

[92] Boelan E.G., Purnomo A.S. (2019). Biodegradation of 1, 1, 1-trichloro-2, 2-bis (4-chlorophenyl) ethane (DDT) by mixed cultures of white-rot fungus Ganoderma lingzhi and bacterium Pseudomonas aeruginosa. HAYATI Journal of Biosciences.

[93] Upadhyay S.K., Rani N., Kumar V., Mythili R., Jain D. (2023). A review on simultaneous heavy metal removal and organo-contaminants degradation by potential microbes: current findings and future outlook. Microbiol. Res..

[94] Bumpus J.A., Aust S.D. (1987). Biodegradation of DDT [1, 1, 1-trichloro-2, 2-bis (4-chlorophenyl) ethane] by the white rot fungus Phanerochaete chrysosporium. Appl. Environ. Microbiol..

[95] Maqbool Z., Hussain S., Imran M., Mahmood F., Shahzad T., Ahmed Z., Azeem F., Muzammil S. (2016). Perspectives of using fungi as bioresource for bioremediation of pesticides in the environment: a critical review. Environ. Sci. Pollut. Res..

[96] Purnomo A.S., Kamei I., Kondo R. (2008). Degradation of 1, 1, 1-trichloro-2, 2-bis (4-chlorophenyl) ethane (DDT) by brown-rot fungi. J. Biosci. Bioeng..

[97] Purnomo A.S., Maulianawati D., Kamei I. (2019).

[98] Schuster E., Dunn-Coleman N., Frisvad J.C., Van Dijck P.W. (2002). On the safety of Aspergillus Niger–a review. Appl. Microbiol. Biotechnol..

